# Evaluating the clinical utility of an easily applicable prediction model of suicide attempts, newly developed and validated with a general community sample of adults

**DOI:** 10.1186/s12888-024-05647-w

**Published:** 2024-03-20

**Authors:** Marcel Miché, Marie-Pierre F. Strippoli, Martin Preisig, Roselind Lieb

**Affiliations:** 1 Department of Psychology, Division of Clinical Psychology and Epidemiology, University of Basel, Missionsstrasse 60-62, 4055 Basel, Switzerland; 2 Psychiatric Epidemiology and Psychopathology Research Center, Lausanne University Hospital, University of Lausanne, Prilly, Switzerland

**Keywords:** Suicide attempt, Clinical utility, Adult, Algorithm, Decision support, Net benefit

## Abstract

**Background:**

A suicide attempt (SA) is a clinically serious action. Researchers have argued that reducing long-term SA risk may be possible, provided that at-risk individuals are identified and receive adequate treatment. Algorithms may accurately identify at-risk individuals. However, the clinical utility of algorithmically estimated long-term SA risk has never been the predominant focus of any study.

**Methods:**

The data of this report stem from CoLaus|PsyCoLaus, a prospective longitudinal study of general community adults from Lausanne, Switzerland. Participants (*N* = 4,097; *M*_age_ = 54 years, range: 36–86; 54% female) were assessed up to four times, starting in 2003, approximately every 4–5 years. Long-term individual SA risk was prospectively predicted, using logistic regression. This algorithm’s clinical utility was assessed by net benefit (NB). Clinical utility expresses a tool’s benefit after having taken this tool’s potential harm into account. Net benefit is obtained, first, by weighing the false positives, e.g., 400 individuals, at the risk threshold, e.g., 1%, using its odds (odds of 1% yields 1/(100-1) = 1/99), then by subtracting the result (400*1/99 = 4.04) from the true positives, e.g., 5 individuals (5-4.04), and by dividing the result (0.96) by the sample size, e.g., 800 (0.96/800). All results are based on 100 internal cross-validations. The predictors used in this study were: lifetime SA, any lifetime mental disorder, sex, and age.

**Results:**

SA at any of the three follow-up study assessments was reported by 1.2%. For a range of seven a priori selected threshold probabilities, ranging between 0.5% and 2%, logistic regression showed highest overall NB in 97.4% of all 700 internal cross-validations (100 for each selected threshold probability).

**Conclusion:**

Despite the strong class imbalance of the outcome (98.8% no, 1.2% yes) and only four predictors, clinical utility was observed. That is, using the logistic regression model for clinical decision making provided the most true positives, without an increase of false positives, compared to all competing decision strategies. Clinical utility is one among several important prerequisites of implementing an algorithm in routine practice, and may possibly guide a clinicians’ treatment decision making to reduce long-term individual SA risk. The novel metric NB may become a standard performance measure, because the a priori invested clinical considerations enable clinicians to interpret the results directly.

**Supplementary Information:**

The online version contains supplementary material available at 10.1186/s12888-024-05647-w.

## Background

Whether a patient with mental health issues may be at risk of attempting suicide is among the questions that await the therapist’s decision in routine clinical practice. Aside from acute risk of a suicide attempt [SA; 1], there is also a long-term risk, for example, within the next 12 months [2] or beyond. The American Psychological Association defines a SA “as a deliberate but unsuccessful attempt to take one’s own life” [3].

The reduction of long-term SA risk requires the identification of individuals who are at increased SA risk, and who may benefit from interventions that aim to reduce SA risk [e.g., 4–7]. The identification method may be questions from a clinical routine suicide risk assessment. These usually focus on acute risk, which we understand as: the current presence of an individual’s suicidal intentions. It may also be an algorithmically supported risk assessment [8], which mainly focuses on long-term risk of a SA, or of suicide [9].

To the best of our knowledge, and although many (56+) published research reports on suicidality (suicide ideation, SA, and suicide) have used so-called supervised machine learning methodologies [e.g., 10], no report has provided empirical evidence that directly relates to questions of the clinical utility of algorithmically estimated SA risk (for a glossary, see Additional File 1). Clinical utility is understood as a benefit after potential harm has been taken into account (e.g., accepting the benefit of a drug despite its side effects). This concept of clinical utility can be applied to individuals and populations [11].

Among the 56 studies included in the meta-analysis by Kusuma et al. [10], three used an adult community sample to prospectively predict SA. One study used Danish national registries as their data source [12] (*N* = 22,974 SA cases, 265,183 controls, SA rate = 8.7%, 1458 candidate predictors); the other two [13, 14] used the National Epidemiologic Survey on Alcohol and Related Conditions (*N* = 34,653 and 32,700, candidate predictors 643 (minimum) and 55, respectively, SA rate in both studies = 0.6%). We found an additional study, where a Korean adult community sample was used to predict the combined outcome of suicide plan/attempt [15] (*N* = 488, SA rate = 50%, 57 candidate predictors). The only result, which has been consistently reported across these studies is, how well, on average, the algorithm can correctly rank an individual who reported the outcome (case) versus an individual who did not report the outcome (non-case). Correct ranking means that the algorithm assigns a higher risk to a case than to a non-case. The closer this average ranking success approaches the value one, the better the algorithm can separate a case from a non-case. Results varied between 0.82 and 0.9 across the four studies. It is important to emphasize that this average ranking success of an algorithm is of very little relevance regarding clinical usefulness [16]. Only Lee and Pak [15] included logistic regression among their selected algorithms, whereas Machado et al. [14] included elastic net, which is an extended way of using logistic regression. However, none of these studies has evaluated the clinical utility of an algorithm that prospectively predicts individual long-term SA risk.

We aimed to evaluate the clinical utility of the logistic regression algorithm, using an adult sample of the general population, and prospectively predict individual long-term SA risk. Based on a meta-analysis of risk factors for suicidal thoughts and behaviors [17], including 365 studies, published between 1965 and 2015, we employed, as predictors, the four SA risk factors, namely, lifetime SA, any lifetime diagnosis of a mental disorder, sex, and age.

## Method

### Study participants

The research data stems from the prospective cohort study CoLaus|PsyCoLaus [18, 19] designed to assess (1) cardiovascular risk factors (CVRFs) and mental disorders in the community, and (2) the associations between CVRFs and mental disorders. CoLaus|PsyCoLaus includes a random sample of 6,734 participants (age range: 35–75 years) selected from the general population according to the civil register of the city of Lausanne (Switzerland) between 2003 and 2006. After a first physical and psychiatric investigation, which took place between 2003 and 2008, the cohort was followed up for approximately 5 (first follow-up, FU1), 9 (second follow-up, FU2), and 13 (third follow-up, FU3) years. At baseline, the psychiatric evaluation was restricted to the 35- to 66-year-old participants in the physical exam, resulting in a 67% participation within this age range (*N* = 3,719). From FU1 on, all individuals from the initial cohort were eligible for psychiatric evaluation. A total of 5,120 participants agreed to at least one psychiatric evaluation. The present study uses data from the 4,097 (54.5% women, age range 35.8–86.6 years) participants who additionally completed at least a second follow-up psychiatric evaluation. Forty-seven participants were excluded because of incomplete data (Sect. 1 in Additional File 2), resulting in a final sample of 4,050 participants.

### Ethics

The institutional Ethics Committee of the University of Lausanne, which afterward became the Ethics Commission of the Canton of Vaud (www.cer-vd.ch), approved the baseline CoLaus|PsyCoLaus study (reference 16/03; 134-03,134-05bis, 134-05-2to5 addenda 1to4). The approval was renewed for the first (reference 33/09; 239/09), second (reference 26/14; 239/09 addendum 2), and third (PB_2018-00040; 239/09 addenda 3to4) follow-ups. The study was performed in agreement with the Helsinki declaration and its former amendments, and in accordance with the applicable Swiss legislation. All participants gave written informed consent.

### Measurements

Sex and age were obtained via participants’ self-report. Mental health information was gathered with the French version [20] of the Diagnostic Interview for Genetic Studies (DIGS) [21], a semi-structured clinical interview assessing symptoms of *DSM-IV-TR* mental disorders [22]. Interrater agreement of the French DIGS was excellent, albeit with a slightly lower 6-week test–retest reliability for psychotic mood disorders [23] and substance use disorders [24]. The DIGS was completed with the anxiety disorders sections of the French version of the Schedule for Affective Disorders and Schizophrenia–Lifetime and Anxiety disorder version [25, 26]. SA history was assessed in a separate interview module, asking whether participants had ever (first evaluation) or since the last interview (follow-up assessments) attempted to end their life. All four risk factors were assessed at the first psychiatric evaluation. The prospective outcome SA was assessed across the three follow-ups. The interviewers were master-level psychologists who were trained over a 1- to 2-month period. Each interview and diagnostic assessment was examined by an experienced psychologist. Participants who confirmed a SA, were asked additional questions related to the SA. Of the total of 48 participants with a SA during follow-up, 32 confirmed the question whether they really wanted to die, while 12 answered no, four participants were not sure. Medical treatment due to the SA was required in 27 of the 48 SAs, 20 negated the question, one participant was not sure. Of 18 recorded suicides in the overall research sample of 6,734 participants, there were three suicide victims among the 4,050 participants analyzed in this study.

### Selection of predictors

The predictors that were a priori selected are lifetime SA, any lifetime mental disorder (major depressive disorder, any anxiety disorder [generalized anxiety disorder, panic disorder, agoraphobia, social phobia], alcohol abuse or dependence, or illicit drug abuse or dependence), sex, and age. The following reasons guided the predictor selection: First, all four predictors are long-known risk factors for SA [17]. Second, these risk factors are already assessed or can easily be assessed in routine clinical practice. Third, we assumed that measurement error in these four predictors is very low [27]. Fourth, the prevalence rates of the binary predictors (lifetime SA, any lifetime mental disorder, sex) are not all low, which is why the heuristic of 10 outcome events per variable (EPV) may yield sufficiently robust prediction model coefficients [28].

### Logistic regression

The logistic regression algorithm was our primary choice for three reasons: firstly, various review articles have concluded that logistic regression is not outperformed by modern machine learning algorithms [e.g., 29–32]; secondly, it is transparent regarding its inner mechanisms (linear algebra); thirdly, its output can be regarded as predicted probability that the outcome was observed, owing to logistic regression being rooted in probability theory.

### Competing algorithm: CART

We selected the classification and regression tree (CART) model [33, 34] to compete against logistic regression. Good statistical practice suggests providing empirical evidence that the analyst’s preferred data model is better, or at least not inferior to, alternative models. CART is a strong competitor, because unlike logistic regression, it automatically makes use of possible interactions between predictors. Furthermore, like logistic regression, CART’s inner mechanisms can be made fully transparent, even to lay users [35]. Because of our limited effective sample size of 48 outcome cases, instead of properly optimizing CART, we conducted a sensitivity analysis, by a posteriori pruning of the decision tree (Sect. 2 in Additional File 2). We used CART’s option of case weights, which corresponded to the harm-to-benefit ratio (explained below, see Clinical utility measure: Net benefit).

### Repeated internal cross-validation

The estimation of real-world clinical utility is based on resampling procedures [36], generally referred to as cross-validation. We used 100 repetitions of holdout resampling, each containing 3,240 individuals (outcome no = 3,202, yes = 38) for training (80%), and 810 individuals (outcome no = 800, yes = 10) for testing (20%). These testing subsets formed the basis for evaluating the model’s clinical utility.

### Software and prediction modeling guidelines

For all analyses and their reporting, including visualization of results, we used the R statistical software environment [37], specifically, the R software packages rpart [38], rms [39], precrec [40], and ggplot2 [41]. Furthermore, we used R code that was provided as an appendix in Austin and Steyerberg [42]. We followed the guidelines for transparent reporting of a multivariable prediction model for individual prognosis or diagnosis (TRIPOD) [43, 44], which we have documented in Sect. 3 of Additional File 2.

### Clinical utility measure: net benefit

Net benefit (NB) is a decision analytic measure, which expresses clinical utility [45, 46]. NB of a decision strategy is defined as providing more than zero true positive individuals, without an increase of false positive individuals. A true positive is defined as a correct prediction, whereas a false positive is defined as a mistaken prediction (i.e., a false alarm). NB is the key result derived from a so-called decision curve analysis (DCA), which asks whether the clinical benefits exceed the expected costs. NB is our main measure of interest in this study. That is, clinical prediction models are developed to help improve clinical treatment decisions. The first important decision, however, is whether a prediction model is of clinical value, which NB can answer directly, as opposed to other commonly reported measures [47, 48].

The clinical utility of using the prediction model to guide treatment decision making competes against two other decision strategies in a DCA, termed “treat none” and “treat all”. Treating all can theoretically prevent the outcome in the entire treated population, at the cost of a possibly very large part of the population being needlessly treated. Conversely, deciding to treat nobody makes any treatment-related benefits or harms impossible. The decision strategy that is of highest clinical value is the one that displays the highest NB, compared to all competitors, e.g., two or more competing prediction models or one prediction model with two or more different predictor sets. Importantly, there are no further criteria, regarding what qualifies as the highest NB, i.e., should two NB results differ by, say, 0.0001, the higher of the two NB results counts as higher.

Before NB can be computed, one must consider how much more important it would be in a clinical setting for a prediction model to correctly predict the outcome (true positive), such as future SA, compared to falsely predicting it (false positive). For instance, clinicians might come to a consensus that for every true positive prediction, there may be 99 false positive predictions. This consensus should be based on the expected benefits of preventing a SA, in contrast to the expected prevention costs (e.g., the resources to treat 99 false positive individuals for each treated true positive individual). Benefits include the noninterrupted participation in life and avoiding costly treatments arising from an attempted suicide. Costs include the intervention’s implications, such as each individual’s involved efforts, the clinic’s or health system’s available resources, and the expected side effects of the intervention.

NB ranges between 0 and 1 and is obtained at a given threshold probability ${p}_{\text{t}}$ (e.g., 0.01, 1%), which always corresponds to a specific harm-to-benefit ratio [e.g., 1:99, or 0.01/(1–0.01)]. For example, an NB of 0.2 means there will be 20 more true positives among 100 tested individuals, without an increase in false positives, compared to treating nobody.

Overall, when assessing the clinical utility of a prediction model against other decision strategies (e.g., treat all) across the a priori selected range of reasonable thresholds, the decision strategy with the highest NB qualifies as the primary source to guide treatment decision making (for a more detailed description of DCA and NB, see Sect. 4 in Additional File 2).

### Delta NB

Delta NB was defined as the additional net increase of true positives at a given ${p}_{\text{t}}$, when using logistic regression and CART, respectively. That is, for ${p}_{\text{t}}$ less than the outcome incidence of 1.23%, each prediction model was compared to the treat all decision strategy, whereas for ${p}_{\text{t}}$ equal to or greater than the outcome incidence, each prediction model was compared to the treat none decision strategy. A negative delta NB indicates that the model may cause more harmful clinical decisions, compared to an alternative decision strategy, e.g., treat all [49], and therefore cannot be recommended for clinical use.

### Reasonable range of threshold probabilities

DCA requires researchers to set a range of reasonable threshold probabilities, accommodating varying threshold preferences across individuals for deciding whether to take outcome-preventing actions [45, 50, 51]. We selected the following threshold probabilities: 0.5%, 0.75%, 1%, 1.25%, 1.5%, 1.75%, and 2%.

Clinically, these low thresholds emphasize the benefit of capturing true positive individuals over the cost of capturing false positive individuals. This trade-off eventually raises legal, ethical, and economic concerns [52], which are beyond the scope of this report. Methodologically, thresholds that are close to, as opposed to being distant from, the outcome rate in the study sample, render the prediction model less sensitive to model miscalibration [49]. This seems reasonable for an initial proof-of-concept report.

### Prediction performance measures

We report the area under the precision-recall curve (PR AUC), the area under the receiver operating characteristic curve (ROC AUC), [40], and the Integrated Calibration Index (ICI; Austin and Steyerberg [42]). All three measures summarize performance across all threshold probabilities. The chance level for the PR AUC is the outcome rate in the validation data (in our study, 0.0123) and 0.5 in the ROC AUC. Perfect discrimination is represented by the value 1 in both the PR AUC and ROC AUC. The ICI shows better calibration the closer it is to 0.

Additional performance results and visualizations are presented in Sect. 5 of Additional File 2. The prediction performance measures are reported only for logistic regression (as CART was selected as a competitor only regarding clinical utility). Performance results for CART are available upon request.

## Results

Table 1 provides the distribution of the predictors used in this report. Major depressive disorder, any anxiety disorder, abuse or dependence of alcohol and illicit drugs, respectively, were merged into lifetime mental disorder, which was then used as one of four predictors. The participants’ ages ranged from 35.8 to 85.6 years (*M* = 53.9 years, *SD* = 11.1). For further information, see Sect. 1 of Additional File 2.

**Table 1:** Distribution of the variables used in this report (*N* = 4,050)

Variable	0		1	
	n	%	n	%
Sex (0 = male, 1 = female)	1,841	45.46	2,209	54.54
Lifetime mental disorder^a^	1,853	45.75	2,197	54.25
Major depressive disorder	2,373	58.59	1,677	41.41
Any anxiety disorder	3,405	84.07	645	15.93
Alcohol abuse or dependence	3,642	89.93	408	10.07
Illicit drug abuse or dependence	3,830	94.57	220	5.43
Lifetime SA	3,845	94.94	205	5.06
Follow-up SA	4,002	98.81	48	1.19

Note. Except for sex, 0 = no and 1 = yes; *n* = number of participants; SA = suicide attempt. ^a^Major depressive disorder, any anxiety disorder (generalized anxiety disorder, panic disorder, agoraphobia, social phobia), alcohol abuse or dependence, illicit drug abuse or dependence

### Logistic regression model coefficients

The estimated model coefficients of the logistic regression model for the full sample (*N* = 4,050) are presented in Table 2. For more detailed results, see Sect. 6 of Additional File 2.

**Table 2:** Logistic regression model coefficients

	Coefficient
Intercept	-4.0296
Lifetime SA	2.5446
Lifetime mental disorder	1.1711
Sex	-0.0631
Age	-0.0423

Note. SA = suicide attempt

### NB and delta NB

**Fig. 1:**
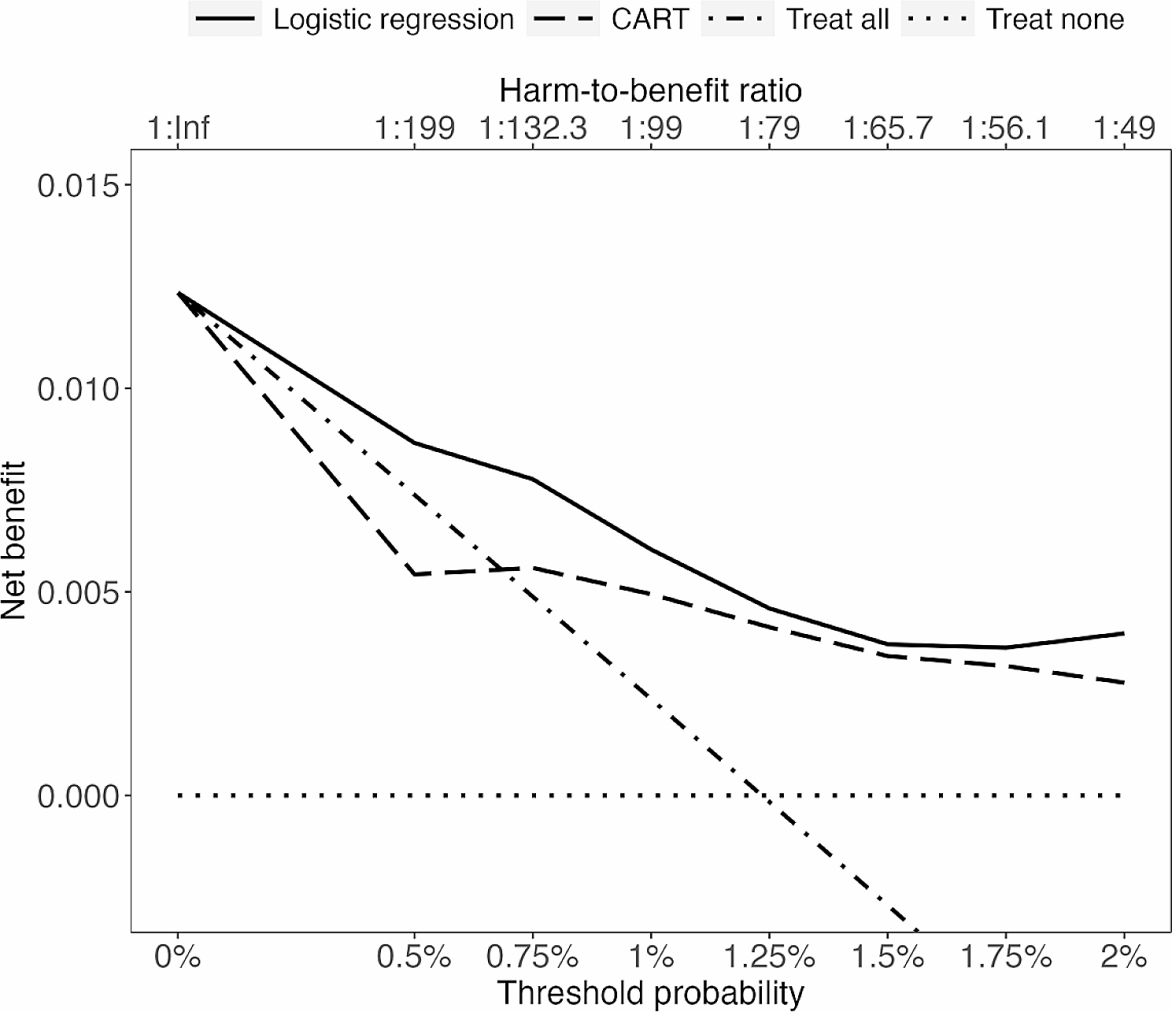
Median net benefit among 100 test subsamples, based on the resampling procedure, which was the same for each of the seven selected threshold probabilities. The solid line shows the logistic regression model, and the dot-dashed line shows the classification and regression tree (CART) model. The harm-to-benefit ratios are rounded to one decimal place (Inf = infinity, expressing an infinite harm to missing any true positive subject in the entire population)

The 100 repetitions of the cross-validated NB for each of the selected threshold probabilities are summarized, using the median NB, in Fig. 1; Table 3 (for detailed results and visualization, see supplementary R package ). Logistic regression showed a somewhat higher median NB across the entire range of thresholds, compared to treat all and treat none. CART showed a lower median NB at the 0.5% threshold, compared to treat all. Across 700 cross-validations, logistic regression indicated 18 times that it was a potentially harmful decision strategy (including 14 times at ${p}_{\text{t}}$ 0.5% and two times at ${p}_{\text{t}}$ 0.75%), compared to 119 times for CART (including 75 times at ${p}_{\text{t}}$ 0.5% and 38 times at ${p}_{\text{t}}$ 0.75%). For more detailed NB results and visualization of all 100 cross-validated decision curves, see Sect. 7 of Additional File 2, especially the instruction and example therein, under the headline Detailed results.

**Table 3:** Net benefit and delta net benefit results, as shown in Fig. 1

${p}_{\text{t}}$ %	Net benefit		Median net benefit across 100 cross-validations			
			Logistic regression		CART	
	Treat all	Treat none	Total	Delta	Total	Delta
0.00	0.0123	0	0.0123	0	0.0123	0
0.50	0.0074	0	0.0087	0.0013	0.0054	-0.0020
0.75	0.0049	0	0.0078	0.0029	0.0056	0.0007
1.00	0.0024	0	0.0060	0.0037	0.0049	0.0026
1.25	< 0	0	0.0046	–	0.0041	–
1.50	< 0	0	0.0037	–	0.0034	–
1.75	< 0	0	0.0036	–	0.0032	–
2.00	< 0	0	0.0040	–	0.0028	–

Note. CART = Classification and regression tree; the column ${p}_{\text{t}}$ % contains the seven selected threshold probabilities; 0% was added to satisfy reporting guidelines for decision curve analysis. The ${p}_{\text{t}}$ of 1.25% is larger than the outcome rate in the test data (0.0123 = 1.23%); yielding a negative net benefit (< 0) for treat all, as do all ${p}_{\text{t}}$ > 1.25%. Empty cells are empty because delta net benefit is equal to net benefit, according to our delta net benefit definition. Lower net benefit results were observed 18 times (logistic regression) and 119 times (CART) across 700 cross-validations, compared to either treat all or treat none

### Logistic regression prediction performance

The PR AUC ranged between 0.028 and 0.436, with 75% of results being below 0.163. Subjects who attempted suicide received a higher predicted probability in 80% of all pairwise comparisons between subjects with vs. subjects without a reported SA (see ROC AUC median in Table 4). Further results, in full detail, such as true positives, false positives, sensitivity, and positive predictive value, can be found in the supplementary R package (see also Sect. 7 of Additional File 2, headline Detailed results).

**Table 4:** Logistic regression prediction performance results across 100 cross-validations

Predictor	Min	1st Qu.	Mean	Median	3rd Qu.	Max
PR AUC	0.028	0.074	0.132	0.106	0.163	0.436
ROC AUC	0.644	0.771	0.805	0.800	0.846	0.916
ICI	0.003	0.006	0.008	0.008	0.010	0.018

Note. Min = minimum; 1st Qu. = 25% quantile; 3rd Qu. = 75% quantile; Max = maximum; PR AUC = area under the precision-recall curve; ROC AUC = area under the receiver operating characteristic curve; for both, a value of 1 represents perfect discrimination; ICI = Integrated Calibration Index; a value of 0 for the ICI represents perfect calibration

## Discussion

We presented the evaluation of the clinical utility of using logistic regression to prospectively predict long-term SA risk in adult individuals of a general community sample. Clinical utility was observed across the entire range of selected threshold probabilities, qualifying logistic regression as the best source to guide clinical decision making, compared to the alternatives of CART, treating everybody, and treating nobody. However, the CART sensitivity analysis indicated that using optimization, CART may qualify as the best decision guide for two of the seven threshold probabilities (1.5% and 1.75%); see Sect. 2 of Additional File 2.

In our investigation we used an extremely parsimonious approach, which is in line with clinically realistic demands. Indeed, Jaccobucci et al. [53] indicated that it may be premature to believe that only lots of predictors can produce clinically useful predictions of a suicidal outcome. It may be that logistic regression with four predictors suffices to predict long-term SA risk, as we have demonstrated for, we believe, the first time. Notably, our approach was parsimonious; for instance, the four predictors are commonly obtained in routine clinical practice, which may greatly facilitate the implementation of such an algorithm in that setting, provided that prediction success can be replicated and that it is continuously supervised once implemented [54].

The parsimonious approach we took may have another advantage. It may maximize the ease of conducting external, as opposed to internal, model validation [55], which is among the most important tasks one should complete prior to model deployment in the real world [56]. External validation could thus be conducted in a large number of independent datasets and then be meta-analyzed, providing empirical arguments for or against large-scale implementation of a SA prediction model in routine clinical care of adults.

The primary reason to consider using a prediction model in clinical practice, despite knowing that false positive predictions will be inevitable, is that it may still improve clinical decision making. This report is the first, in the realm of SAs, to have focused on NB. One of the most important prerequisites of NB is to present an idea of what the clinical decision may entail, because only then can a reasonable range of threshold probabilities be agreed upon across researchers, clinicians, and other stakeholders.

As research of clinical prediction models of SAs in adults progresses [8], it will be important that other researchers also present clinically feasible models, e.g., open box, as opposed to black box, prediction models, containing few candidate predictors which are easy to assess in clinical practice. Should the NB, if presented in a sufficient number of studies, indicate clinical utility across a reasonable range of risk thresholds, discussing clinical implementation is warranted. This recommendation statement [57] and the criticism of it [58] are current examples of discussing implementation of screening instruments, in a population wide effort of preventing depression and suicide in US adults. The range of reasonable risk thresholds depend on the evaluation of attempted suicide, which we evaluate as a very serious outcome, and the clinical action to be provided to individuals whose estimated risk exceeds the risk threshold. For instance, more extensive diagnostic procedures may justify a wide dissemination, due to relatively low costs, as opposed to a therapeutic intervention, due to relatively high costs. Wide dissemination means a high number of false positive individuals, e.g., 99, for each true positive individual may clinically be acceptable, i.e., 1:99 (risk threshold of 1%). However, such matters must be discussed and agreed upon by all involved stakeholders, who belong to diverse groups, such as therapists, patients, public health politicians, and lawyers.

Eventually, should official public health institutions approve the use of a SA prediction model in clinical practice, its use must be very simple. That is, both the therapist and the patient must be able to use the risk algorithm and interpret the result effortlessly. For example, a man, 51 years old, having reported a lifetime suicide attempt, but having no lifetime mental disorder diagnosis, receives an estimated risk of a future SA of 2.5%, using our logistic regression model, which is presented in Table 2 (the model is published as part of the supplementary R package ). Since 2.5% exceeds the maximum value of our suggested reasonable range of risk thresholds (0.5–2%), the therapist would offer this man the preventive action, e.g., more extensive SA diagnostics.

Overall, visual presentation, a clear interpretation, and a transparent explanation of the individual’s estimated SA risk is warranted, which includes communicating to the individual the uncertainty of his or her estimated risk [59]. Some authors suggest the use of so-called nomograms [60, 61], which may facilitate the use of risk algorithms in clinical practice. A nomogram is a graphical representation of a mathematical formula, which is what a prediction model is. Such future possibilities require substantial amounts of clinically relevant research.

### Strengths

First, we followed recommendations against using any of the up or downsampling methods for logistic regression [62]. Second, we used only four candidate predictors, which are already assessed in routine clinical care. Third, we present our complete analysis code, as well as the logistic regression prediction model (supplementary R package), which can be downloaded from this GitHub repository https://github.com/mmiche/predictSuiattPsyCoLaus. Although we cannot publish the original CoLaus|PsyCoLaus study data, we provide code to simulate data, which is superficially similar to the original study data.

### Limitations

First, we had a very small effective sample size (*N* = 48 outcome cases). However, by using four predictors, we met the EPV heuristic of approximately 10 outcome cases per predictor in the training subsample. Second, we used a single question to measure the outcome SA, instead of employing additional qualifiers, such as the intention to die of the person attempting suicide. In our view, it appears justified to regard all participants who affirm this question as constituting a clinically homogeneous and relevant group. Third, we used internal validation, which is the minimum requirement for prediction modeling research [63]. However, this is the first proof-of-concept paper describing such a parsimonious approach to predict SA in a general community adult sample, which is why we think internal validation is a justifiable limitation. Note that Sect. 8 of Additional File 2 permits readers to judge for themselves whether overfitting was strong.

## Conclusion

Despite the strong class imbalance of the outcome (98.8% no, 1.2% yes) and only four predictors, clinical utility (number of true positives greater than the weighted number of false positives) was observed across the full range of reasonable risk thresholds. If comparable future research also indicated clinical benefit of an algorithm’s estimates of adult SA risk, clinician’s routine use of a risk algorithm would improve their decision making overall, by using the tool that was empirically better than all other tested clinical decision strategies. This may eventually help save lives or prevent individuals from attempting suicide.

### Electronic supplementary material

Below is the link to the electronic supplementary material.

Additional File 1: Glossary of terms, used in the main document.

Additional File 2: Additional information, such as a detailed explanation of decision curve analysis, results in more detail, and sensitivity analyses.

## Data Availability

Yes, Additional File 2 and the supplementary R package. The supplementary R package can be downloaded from this GitHub repository https://github.com/mmiche/predictSuiattPsyCoLaus. Although we cannot publish the original CoLaus|PsyCoLaus study data, we provide code to simulate data, which is superficially similar to the original study data.
