# Supplementary material for: Evaluating the clinical utility of an easily applicable prediction model of suicide attempts, newly developed and validated with a general community sample of adults
Source: BMC Psychiatry. 2024 Mar 20;24:217. doi: 10.1186/s12888-024-05647-w (PMC10953234; doi:10.1186/s12888-024-05647-w)
Supplement: Supplementary file 1 — Additional File 1: Glossary of terms, used in the main document. [file 12888_2024_5647_MOESM1_ESM.docx]

**Additional File 1.**

Glossary of terms, used in the main document.

**Prediction model**: In a clinical context, an outcome is very often dichotomous, e.g., a disease either will or will not develop. Prediction models are used to estimate the risk of an individual to developing a certain outcome, which may be a disease, a mental disorder, or some other clinically relevant outcome. The prediction model estimates the risk by using measured values of health indicators which are deemed predictive of the outcome. The estimated risk is often expressed as a percentage (values between 0 and 100). If this estimated risk is above a certain cutoff, which can but does not have to be 50, the individual is classified by the prediction model to be at an increased risk to develop the outcome. If the estimated risk is equal to or below the cutoff, the individual is considered not to be at an increased risk.

**Model coefficients**: A prediction model is a mathematical formula. If it is combined with numbers, a result will be produced, e.g., a predicted outcome value. Every undergraduate psychology student has been exposed to the formula of the simple linear regression model: $y=a+b*x$, with $y$ being the predicted (continuous) outcome value, $a$ being the model intercept, $b$ being the regression weight, and $x$ being the placeholder for a predictor value. Model coefficients, in this example, refer to the intercept and the regression weight. If the model coefficients are real numbers, any numeric predictor value can be used to estimate (to predict) the outcome value. The main differences to the prediction models of our study are that the observed outcome is dichotomous, instead of continuous, and that it contains four instead of a single predictor.

**Logistic regression**: Logistic regression can be used for different purposes, of which prediction is one. As with linear regression (see Model coefficients above), logistic regression can be combined with data, often termed fitting the model to data, which assigns real numbers to the model coefficients. When applying this prediction model to new data, one of the model results is the predicted probability of the dichotomous (see Prediction model above) outcome to be present. Probabilities range between 0 and 1.

**Candidate predictors**: A prediction model (see Prediction model above) may contain one or more predictors. The term “candidate predictors” is used in opposition to simply “predictors”, given that there are important rules that must guide the number of predictors present within a model. If these rules are violated, the model coefficients (see Model coefficients above) can become unstable. One important rule is the event per variable (EPV) ratio to be sufficiently high, e.g., 10, 20, or even higher. That is, there should be, for example, 10 outcome cases available for each predictor in the model. If a prediction model contains, say, 30 predictors, there should be at least 300 outcome cases in the data, for an EPV ratio of 10. If from that initial model, 10 predictors were removed, then still, the 30 predictors are the candidate predictors.

**Cross-validation** (CV): CV refers to producing a prediction model with one group, then to apply the produced model to another group. If the health indicators, which are used as predictors in the model, are predictive in one particular group, they should also be predictive in other groups, provided that all single groups belong to the same overall target population of the prediction model. CV aims to find out whether a prediction model is predictive in different groups, as opposed to just one or very few specific groups from the population.

**Repeated internal CV**: Internal CV differs from external CV by how independent the group is, which was used to produce the prediction model, from the group, to which this prediction model is then applied. If the total sample originates from a single study, it must be split into at least two parts, this is done so as to cross-validate the prediction model. Both resulting subsamples are dependent, because they originate from the same study, which is why it is termed an “internal CV”. If the prediction model is produced in one sample, and then applied to an independent other sample, it is termed an “external CV”. Internal CV can be repeated by repeatedly using a random subset of the total sample to produce the prediction model, after which the remaining subset of the total sample is used to apply the prediction model to. This repeated internal CV is supposed to give an impression of how robust the predictions are.

**Risk threshold**: As mentioned in the description of a prediction model, the estimated risk is often expressed as a percentage. This corresponds to the concept of risk being a probability. Probabilities are defined as ranging between 0 and 1. Depending on certain considerations, one might set the risk threshold to a low value, say, 0.1 (or 10%, if expressed as percentage). All individuals whose estimated risk is above 10% would therefore be “detected” as being of increased risk to develop the outcome. A low-risk threshold indicates that it is very important to detect many individuals who will actually develop the outcome (termed true positives), at the cost of falsely labeling a possibly large number of individuals as being of increased risk to develop the outcome (termed false positives). Prediction models can of course never predict an outcome perfectly, which is why the tradeoff between true and false positives must be discussed and agreed upon, in order to use a prediction model for socially critical purposes, such as mental health outcomes.

**Prediction performance measure**: There is a plethora of measures with which to evaluate the accuracy of a prediction model in predicting the outcome within a validation sample. This is often termed “performance” of a prediction model. Which measure or measures are presented to readers, should, in our view, be guided by the ultimate research aim. If this aim is clinical implementation of the prediction model, then primarily a measure is warranted that permits the evaluation of the clinical value of the prediction model, such as net benefit.
