# Supplementary material for: Evaluating the clinical utility of an easily applicable prediction model of suicide attempts, newly developed and validated with a general community sample of adults
Source: BMC Psychiatry. 2024 Mar 20;24:217. doi: 10.1186/s12888-024-05647-w (PMC10953234; doi:10.1186/s12888-024-05647-w)
Supplement: Supplementary file 2 — Additional File 2: Additional information, such as a detailed explanation of decision curve analysis, results in more detail, and sensitivity analyses. [file 12888_2024_5647_MOESM2_ESM.docx]

**Additional file 2.**

This document is a supplement to the article “Evaluating the clinical utility of an easily applicable prediction model of suicide attempts, newly developed and validated with a general community sample of adults.”

[**Section 1 - Study sample details** 2](#_Toc146030628)

[**Section 2 - CART sensitivity analysis** 4](#_Toc146030629)

[**Section 3 - TRIPOD** 7](#_Toc146030630)

[**Section 4 - DCA explanation** 10](#_Toc146030631)

[**Section 5 - Calibration details** 16](#_Toc146030632)

[**Section 6 - Logistic regression model output** 20](#_Toc146030633)

[**Section 7 - DCA details** 21](#_Toc146030634)

[**Section 8 - Development samples** 24](#_Toc146030635)

[**References** 28](#_Toc146030636)

# **Section 1 - Study sample details**

Tables S1–S3 provide a more detailed description of the study sample. Tables S1 and S2 are more detailed insofar as the single diagnostic groups are shown, which are subsumed in “lifetime mental disorder”, which is one of the four predictors in our prediction model. See main article, Method section, Study participants, and Results section, Table 1.

**Table S1**. Distribution of variables in the full study sample (*N* = 4,097)

| Variable | 0 | | 1 | | Total (*N*) | Missing data |
| --- | --- | --- | --- | --- | --- | --- |
|  | *n* | % | *n* | % |  |  |
| Sex (0 = male,  1 = female) | 1,864 | 45.50 | 2,233 | 54.50 | 4,097 | 0 |
| Lifetime mental disorder^a^ | 1,875 | 45.77 | 2,222 | 54.23 | 4,097 | – |
| Major depressive disorder | 2,400 | 58.58 | 1,697 | 41.42 | 4,097 | 0 |
| Any anxiety disorder^b^ | 3,449 | 84.18 | 648 | 15.82 | 4,097 | 0 |
| Alcohol abuse or dependence | 3,673 | 89.91 | 412 | 10.09 | 4,085 | 12 |
| Illicit drug abuse or dependence | 3,868 | 94.57 | 222 | 5.43 | 4,090 | 7 |
| Lifetime SA | 3,867 | 94.94 | 206 | 5.06 | 4,073 | 24 |
| Follow-up SA | 4,030 | 98.82 | 48 | 1.18 | 4,078 | 19 |

*Note*. Except for sex, 0, = no and 1 = yes; *n* = number of participants; SA = suicide attempt.

^a^Major depressive disorder, any anxiety disorder, alcohol abuse or dependence, illicit drug abuse or dependence.

^b^Generalized anxiety disorder, panic disorder, agoraphobia, social phobia.

**Table S2**. Distribution of variables in the reduced study sample (*N* = 4,050)

| Variable | 0 | | 1 | |
| --- | --- | --- | --- | --- |
|  | *n* | % | *n* | % |
| Sex (0 = male, 1 = female) | 1,841 | 45.46 | 2,209 | 54.54 |
| Lifetime mental disorder^a^ | 1,853 | 45.75 | 2,197 | 54.25 |
| Major depressive disorder | 2,373 | 58.59 | 1,677 | 41.41 |
| Any anxiety disorder^b^ | 3,405 | 84.07 | 645 | 15.93 |
| Alcohol abuse or dependence | 3,642 | 89.93 | 408 | 10.07 |
| Illicit drug abuse or dependence | 3,830 | 94.57 | 220 | 5.43 |
| Lifetime SA | 3,845 | 94.94 | 205 | 5.06 |
| Follow-up SA | 4,002 | 98.81 | 48 | 1.19 |

*Note*. Except for sex, 0 = no and 1 = yes; *n* = number of participants; SA = suicide attempt.

^a^Major depressive disorder, any anxiety disorder, alcohol abuse or dependence, illicit drug abuse or dependence.

^b^Generalized anxiety disorder, panic disorder, agoraphobia, social phobia.

**Table S3**. Distribution of age (in years) in the full and reduced study samples

| Sample | Mean | *SD* | Median | Min | Max | Diff | *SE* |
| --- | --- | --- | --- | --- | --- | --- | --- |
| Full (*N* = 4,097) | 53.91 | 11.11 | 52.49 | 35.84 | 86.62 | 50.78 | 0.17 |
| Reduced (*N* = 4,050) | 53.89 | 11.09 | 52.49 | 35.84 | 85.57 | 49.73 | 0.17 |

*Note*. Min = minimum; max = maximum; Diff = Difference between Max and Min age.

Tables S1-S3 show that the descriptive results between the full (N = 4097) and the reduced sample (N = 4050) change very slightly.

# **Section 2 - CART sensitivity analysis**

The reason why we conducted a sensitivity analysis with the classification and regression tree (CART) model was that CART provides options with which to optimize the prediction performance, whereas logistic regression does not provide such options. We used an option called pruning. The software documentation of the R package rpart [1] describes pruning as recursively snipping off the least important splits of the fitted decision tree, based on the complexity parameter *c*_p_. We set five *c*_p_ values from *c*_p_ = 0.01 to *c*_p_ = 0.05.


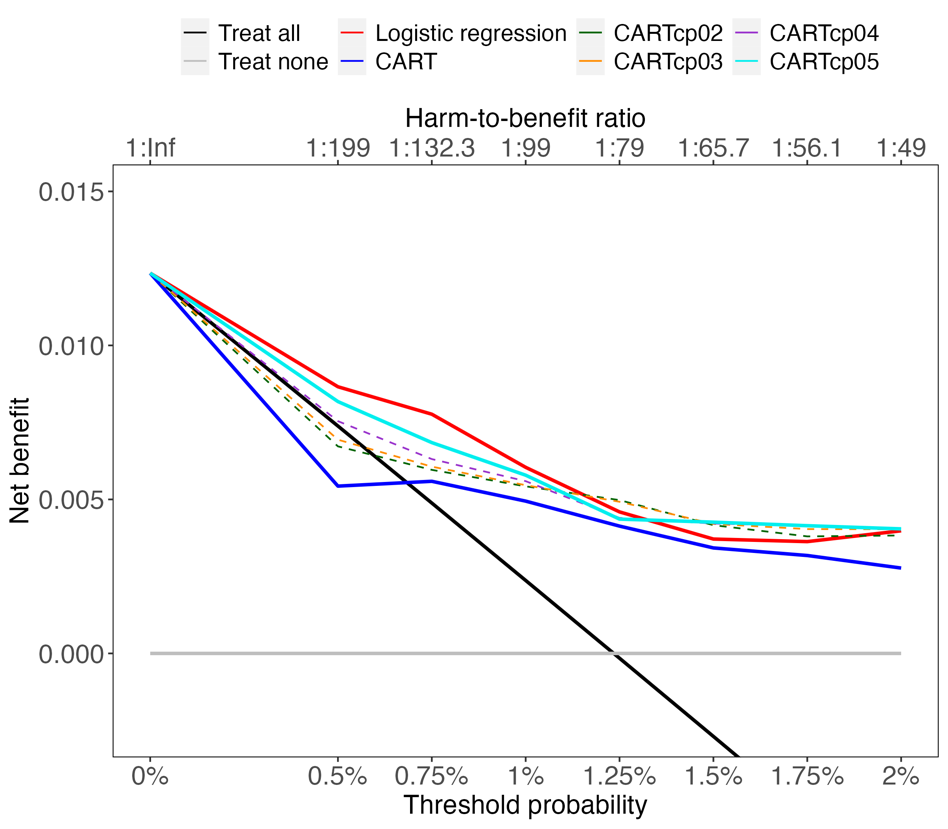


**Figure S1**. Decision curves for decision strategies treat all and treat none, and for six prediction models: Logistic regression (red solid), default CART (blue solid), and pruned CART (complexity parameter *c*_p_ between 0.02 and 0.05). Pruned CART curves for *c*_p_ = 0.02, 0.03, and 0.04 are shown as thin dashed curves between the solid blue and the solid cyan curve. The pruning for *c*_p_ = 0.01 is not shown because there is no difference between this and no pruning.

Pruning may reduce the overfit of the default CART model, therefore increasing chances that pruned CART will predict new data more accurately (which is the main goal of predictive modeling), compared to the default CART model. This can be seen in Figure S1. The blue solid curve is the default CART model (no pruning, *c*_p_ = 0), whereas the solid cyan curve (*c*_p_ = 0.05) shows that the pruned CART has a higher net benefit than the default CART. For the threshold probabilities of 1.5% and 1.75%, pruned CART (*c*_p_ = 0.05) has a higher net benefit (NB) than logistic regression, whereas at the 2% threshold both prediction models have the same NB (0.004). For detailed results see Table S4.

**Table S4**. Detailed net benefit results of the CART sensitivity analysis (median, as shown in Figure S1, maximum, and minimum). For comparative reasons, the logistic regression net benefits are also shown

| *p*_t_ % | Median | Max | Min |
| --- | --- | --- | --- |
| Logistic regression | | | |
| 0.00 | 0.0123 | 0.0123 | 0.0123 |
| 0.50 | 0.0087 | 0.0100 | 0.0050 |
| 0.75 | 0.0078 | 0.0095 | 0.0044 |
| 1.00 | 0.0060 | 0.0093 | 0.0023 |
| 1.25 | 0.0046 | 0.0093 | 0.0006 |
| 1.50 | 0.0037 | 0.0083 | 0.0004 |
| 1.75 | 0.0036 | 0.0078 | -0.0002 |
| 2.00 | 0.0040 | 0.0078 | 0.0003 |
| Default CART (*c*_p_ = 0) | | | |
| 0.00 | 0.0123 | 0.0123 | 0.0123 |
| 0.50 | 0.0054 | 0.0099 | 0.0014 |
| 0.75 | 0.0056 | 0.0093 | 0.0020 |
| 1.00 | 0.0049 | 0.0087 | 0.0014 |
| 1.25 | 0.0041 | 0.0082 | **0.0009** |
| 1.50 | 0.0034 | 0.0080 | **0.0007** |
| 1.75 | 0.0032 | **0.0081** | **0.0004** |
| 2.00 | 0.0028 | 0.0069 | 0.0001 |
| Pruned CART (*c*_p_ = 0.02) | | | |
| 0.00 | 0.0123 | 0.0123 | 0.0123 |
| 0.50 | 0.0067 | **0.0106** | 0.0028 |
| 0.75 | 0.0060 | **0.0098** | 0.0018 |
| 1.00 | 0.0054 | 0.0088 | 0.0012 |
| 1.25 | **0.0050** | 0.0085 | **0.0011** |
| 1.50 | **0.0042** | 0.0079 | **0.0006** |
| 1.75 | **0.0038** | 0.0074 | **0.0005** |
| 2.00 | 0.0038 | 0.0072 | **0.0004** |
| Pruned CART (*c*_p_ = 0.03) | | | |
| 0.00 | 0.0123 | 0.0123 | 0.0123 |
| 0.50 | 0.0069 | **0.0102** | 0.0035 |
| 0.75 | 0.0061 | **0.0098** | 0.0021 |
| 1.00 | 0.0055 | 0.0090 | 0.0008 |
| 1.25 | **0.0049** | 0.0085 | **0.0007** |
| 1.50 | **0.0042** | 0.0083 | **0.0006** |
| 1.75 | **0.0040** | **0.0089** | **0.0005** |
| 2.00 | 0.0040 | **0.0091** | **0.0004** |
| Pruned CART (*c*_p_ = 0.04) | | | |
| 0.00 | 0.0123 | 0.0123 | 0.0123 |
| 0.50 | 0.0075 | 0.0096 | 0.0039 |
| 0.75 | 0.0063 | 0.0089 | 0.0025 |
| 1.00 | 0.0056 | 0.0090 | 0.0008 |
| 1.25 | 0.0044 | 0.0074 | 0.0007 |
| 1.50 | **0.0042** | **0.0083** | **0.0006** |
| 1.75 | **0.0041** | **0.0092** | **0.0005** |
| 2.00 | 0.0040 | **0.0091** | **0.0004** |
| Pruned CART (*c*_p_ = 0.05) | | | |
| 0.00 | 0.0123 | 0.0123 | 0.0123 |
| 0.50 | 0.0082 | 0.0096 | 0.0039 |
| 0.75 | 0.0068 | 0.0089 | 0.0025 |
| 1.00 | 0.0058 | 0.0081 | 0.0008 |
| 1.25 | 0.0044 | 0.0072 | **0.0007** |
| 1.50 | **0.0043** | **0.0083** | **0.0006** |
| 1.75 | **0.0041** | **0.0092** | **0.0005** |
| 2.00 | 0.0040 | **0.0091** | **0.0004** |

*Note*. CART = Classification and regression tree (CART) model. *c*_p_ = complexity parameter; *p*_t_ % = threshold probabilities shown in Figure S1 (*x* axis). Bold numbers indicate higher net benefit compared to the net benefit for logistic regression.

# **Section 3 - TRIPOD**

We downloaded the “TRIPOD Checklist: Prediction Model Development” from the TRIPOD website (URL: <https://www.tripod-statement.org/>), deleted the “Page” column, and then checked each of the listed items; see our blue comments in the TRIPOD checklist below.

**Table S5**. TRIPOD Checklist for prediction model development

| **Section/Topic** | **Item** | **Checklist Item** |
| --- | --- | --- |
| **Title and abstract** | | |
| Title | 1 | Identify the study as developing and/or validating a multivariable prediction model, the target population, and the outcome to be predicted. Yes, done. |
| Abstract | 2 | Provide a summary of objectives, study design, setting, participants, sample size, predictors, outcome, statistical analysis, results, and conclusions. Yes, done. |
|  |  |  |
| **Introduction** | | |
| Background and objectives | 3a | Explain the medical context (including whether diagnostic or prognostic) and rationale for developing or validating the multivariable prediction model, including references to existing models. |
|  |  | *Prospective prediction of suicide attempt is prognostic. Rationale: Currently, there is no research that directly provided empirical evidence of the clinical utility of algorithmically estimated long-term individual SA risk. Also, there are no comparable existing models that use only four predictors that are routinely assessed in clinical practice.* |
|  | 3b | Specify the objectives, including whether the study describes the development or validation of the model or both. |
|  |  | *Objective: Evaluate the clinical utility of the model. The study describes both model development and (internal) validation.* |
|  |  |  |
| **Section/Topic** | **Item** | **Checklist Item** |
| **Methods** | | |
| Source of data | 4a | Describe the study design or source of data (e.g., randomized trial, cohort, or registry data), separately for the development and validation data sets, if applicable. |
|  | 4b | Specify the key study dates, including start of accrual; end of accrual; and, if applicable, end of follow-up. |
| Participants | 5a | Specify key elements of the study setting (e.g., primary care, secondary care, general population) including number and location of centres. |
|  | 5b | Describe eligibility criteria for participants. |
|  |  | *4a, 4b, 5a, and 5b: Main text ("Study participants").* |
| Outcome | 6a | Clearly define the outcome that is predicted by the prediction model, including how and when assessed. |
|  | 6b | Report any actions to blind assessment of the outcome to be predicted. |
| Predictors | 7a | Clearly define all predictors used in developing or validating the multivariable prediction model, including how and when they were measured. |
|  | 7b | Report any actions to blind assessment of predictors for the outcome and other predictors. |
|  |  | 6a, 6b, 7a, and 7b: Main text ("Selection of predictors" and "Measurements"). |
| Sample size | 8 | Explain how the study size was arrived at. |
| Missing data | 9 | Describe how missing data were handled (e.g., complete-case analysis, single imputation, multiple imputation) with details of any imputation method. |
|  |  | 8 and 9: Main text ("Study participants"). |
| Statistical analysis methods | 10a | Describe how predictors were handled in the analyses. |
|  | 10b | Specify type of model, all model-building procedures (including any predictor selection), and method for internal validation. |
|  | 10d | Specify all measures used to assess model performance and, if relevant, to compare multiple models. |
|  |  | 10a, 10b, and 10c: Main text ("Logistic regression", "Competing algorithm: CART", "Repeated internal cross-validation", "Clinical utility measure: Net benefit", "Delta NB", and "Prediction performance measures"). |
| Risk groups | 11 | Provide details on how risk groups were created, if done. See items 7a and 7b. |
|  |  |  |
| **Section/Topic** | **Item** | **Checklist Item** |
| **Results** | | |
| Participants | 13a | Describe the flow of participants through the study, including the number of participants with and without the outcome and, if applicable, a summary of the follow-up time. A diagram may be helpful. |
|  |  | Main text ("Study participants", no diagram provided). |
|  | 13b | Describe the characteristics of the participants (basic demographics, clinical features, available predictors), including the number of participants with missing data for predictors and outcome. |
|  |  | Main text ("Table 1") and Additional File 1 ("Study sample details"). |
| Model development | 14a | Specify the number of participants and outcome events in each analysis. |
|  |  | Main text ("Repeated internal cross-validation"). |
| Model specification | 15a | Present the full prediction model to allow predictions for individuals (i.e., all regression coefficients, and model intercept or baseline survival at a given time point). |
|  |  | Main text ("Logistic regression model coefficients") and supplementary R package documentation: "PsyCoLausSApredictionModel". |
| Model performance | 16 | Report performance measures (with CIs) for the prediction model. |
|  |  | Main text ("NB and delta NB", "Figure 1", "Table 3", "Table 4") and Additional File 1, "Calibration details", "DCA details", "CART sensitivity analysis", and supplementary R package documentation: "predictSuiattPsyCoLaus". No CIs provided. |
|  |  |  |
| **Section/Topic** | **Item** | **Checklist Item** |
| **Discussion** | | |
| Limitations | 18 | Discuss any limitations of the study (such as nonrepresentative sample, few events per predictor, missing data). |
|  |  | Main text (" Study participants" and "Limitations"). |
| Interpretation | 19b | Give an overall interpretation of the results, considering objectives, limitations, and results from similar studies, and other relevant evidence. Yes, done. |
| Implications | 20 | Discuss the potential clinical use of the model and implications for future research. Yes, done. |
|  |  |  |
| **Other information** | | |
| Supplementary information | 21 | Provide information about the availability of supplementary resources, such as study protocol, Web calculator, and data sets. |
|  |  | Additional File 1 and supplementary R package. |
| Funding | 22 | Give the source of funding and the role of the funders for the present study. |
|  |  | No particular funding for the present study. |

*Note*. Item 17 is missing, because it is concerned only with model updating, not with model development.

# **Section 4 - DCA explanation**

This section provides an explanation of decision curve analysis (DCA) and its key metric, net benefit (NB).


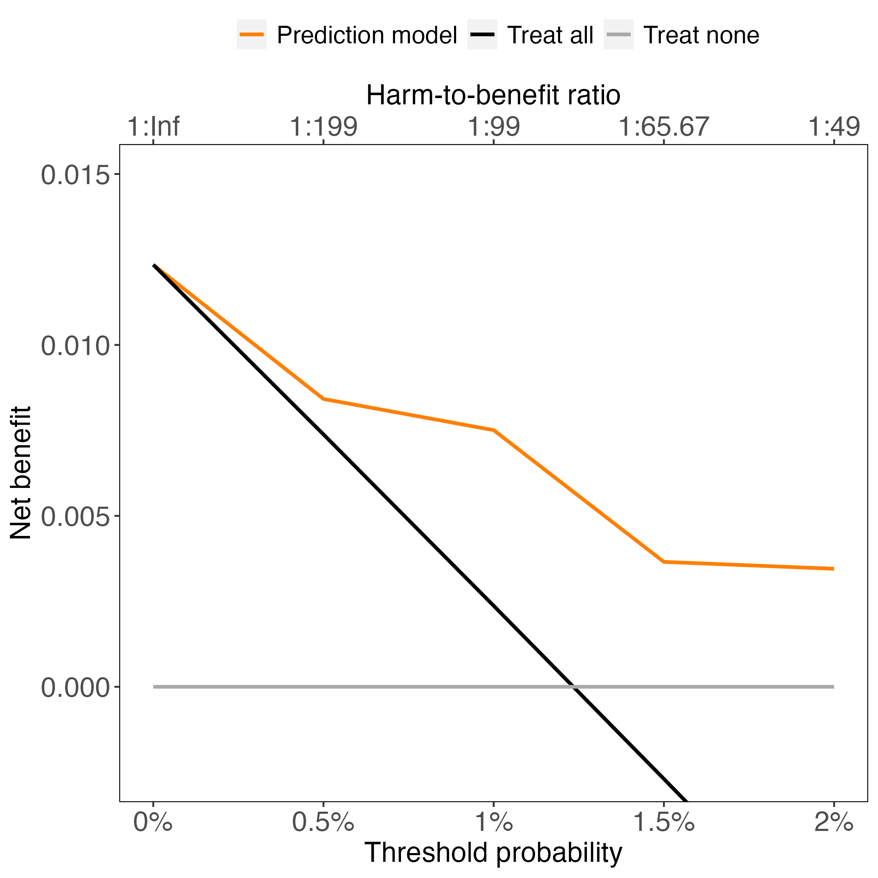


**Figure S2**. Decision curves of three conceivable clinical strategies: an empirical prediction model (orange line), treat all individuals (black line), and treat no individual (gray line; net benefit constantly being zero). The odds of the threshold probability (bottom) yield the harm-to-benefit ratio (top), for example, 2% = odds(0.02) = 0.02/(1–0.02) = 2/98 = 1/49. A harm-to-benefit ratio of 1:49 means that to prevent the outcome in one true positive individual, it is considered worthwhile to treat 49 false positive individuals.

**Clinical relevance**

DCA is used to empirically evaluate the best clinical strategy for deciding whether an individual should receive treatment to help prevent a certain outcome, such as a future suicide attempt. We use suicide attempt as an exemplary outcome throughout this section to explain DCA.

**NB**

The general idea of NB is to express whether a predictive instrument provides more correct than incorrect prognoses, while taking into consideration that a correct prognosis is clinically more important than a false prognosis, that is, whether more true positive predictions remain after having subtracted unavoidable false positive predictions. Indeed, when using an instrument to make clinical prognoses, it will always produce true positive and false positive prognoses. Knowing that a positive prognosis leads to certain treatment decisions in clinical practice, NB aims to answer whether using this instrument is nonetheless the clinically best guide for treatment decision making, when comparing this instrument to alternative decision strategies. For instance, in Figure S2, NB is the highest for the prediction model (an instrument that can make clinical prognoses) across the shown range of threshold probabilities, compared to the two alternative decision strategies treat all and treat none. This makes the prediction model the clinically best guide for treatment decision making.

**Estimating individual risk of a future outcome and prediction errors**

True individual risk probabilities are rarely, if ever, known and therefore have to be estimated. For example, in suicide attempts, the risk for an individual (say, Tom, 30 years old) can be estimated from a suicide attempt prediction model that has been produced using a sample (which does not include Tom) from the same population of which Tom is a member.

Owing to various challenges when estimating individual suicide attempt risk, for example, a rare outcome with possibly many complex causes, every prediction model will make prediction errors. That is, relatively many individuals will be estimated to be at risk of a suicide attempt who, irrespective of having received treatment, would not go on to attempt suicide (false positive predictions). On the other hand, among all individuals who are estimated to be at risk, there may be many who would go on to attempt suicide unless they received timely and adequate treatment (true positive predictions).

In such a clinical scenario, it is important to discuss how many false positive individuals may be treated for each true positive individual whose outcome the treatment may prevent. The outcome as well as the ensuing treatment (e.g., treatment costs, possible adverse effects) strongly determine this discussion. Suicide attempt is a very serious outcome, which is why preventing one suicide attempt may be considered worth the cost of treating a high number of false positives. The treatment may differ in terms of cost and possible adverse effects; for example, therapeutic treatment of suicidality usually differs from administering more in-depth diagnostic suicidality assessment.

**Weighing benefit against harm**

To weigh true positives against false positives is the core tenet of DCA and of NB as its key metric. For instance, in Figure S2, if it had been determined that 99 false positive individuals may be treated for each true positive individual, then the 1% probability threshold would be recommended in clinical practice as the treatment decision cut-off. That is, every individual with a predicted probability of 1% or higher would be eligible for treatment, whereas individuals with a predicted probability of less than 1% would not be eligible. DCA, as shown in Figure S2, indicates that for the threshold probability of 1%, the prediction model provides the empirically best guide for making the clinical treatment decision, because the NB of the prediction model is higher than the remaining decision strategies treat all and treat none (see Figure S2 and Table S6).

**Table S6**. The results that are displayed in Figure S2, except for treat none, which is always zero across all possible threshold probabilities

| *p*_t_ % | Harm-to-benefit ratio | TP | FP | Net benefit | | Treat none |
| --- | --- | --- | --- | --- | --- | --- |
|  |  |  |  | Prediction model | Treat all |  |
| 0.00 | – | 10 | 0 | 0.0123 | 0.0123 | 0 |
| 0.50 | 199 | 9 | 434 | 0.0084 | 0.0074 | 0 |
| 1.00 | 99 | 9 | 289 | 0.0075 | 0.0024 | 0 |
| 1.50 | 65.67 | 5 | 134 | 0.0037 | -0.0027 | 0 |
| 2.00 | 49 | 4 | 59 | 0.0035 | -0.0078 | 0 |

*Note*. *N* = 810. *p*_t_ = threshold probability; TP = true positives; FP = false positives; outcome incidence = 10/810 = 0.0123 (1.23%).

**Understanding the “treat all” curve and computing its NB**

Treating all is the same as assuming all individuals in the population are positive, that is, that everybody will develop the specified outcome if they do not receive treatment. The fraction of the population for whom this is true may benefit from the treat all strategy. Therefore, the outcome might thus be reduced to zero. The gradual reduction of the risk of developing the outcome is represented by the treat all curve. This logic explains why NB reaches 0 when the treat all curve crosses the threshold probability axis at the outcome incidence. That is, all individuals in the population who had increased outcome risk have been successfully treated; therefore, the NB of this decision strategy must be negative for all remaining individuals in the population, who have no increased outcome risk. The negative NB of the treat all curve represents harms and/or costs, such as the time spent being treated unnecessarily. Computationally, the treat all curve results from

$$outcome incidence-\left( \frac{p_{t}}{1-p_{t}} \right)*\left( 1-outcome incidence \right),$$

where $\left( \frac{p_{t}}{1-p_{t}} \right)$ is the harm-to-benefit ratio at the threshold probability $p_{t}$.

For instance, the treat all NB at the 1% threshold of 0.0024 (see Table S6) results from

$$0.0123-\left( \frac{0.01}{1-0.01} \right)*\left( 1-0.0123 \right).$$

The outcome incidence (not rounded) is equal to 10/810. The gradually decreasing treat all curve is always perfectly smooth, owing to how it gets computed.

**Understanding the prediction-model curve and computing its net benefit**

As described above, data from a population sample can be used to develop a prediction model. For instance, the logistic regression model returns predicted outcome probabilities, that is, each individual’s probability of having the outcome, as predicted by the logistic regression model, based on the validation data set.

The prediction-model curve shows the NBs that result for each of various cut-offs across the selected threshold probabilities. As discussed above, the highest overall NB qualifies as the clinically best guide for treatment decision making. Computationally, for a given threshold probability $p_{t}$, the prediction model curve is produced by this formula:

$$\frac{True Positives}{n}-\frac{False Positives}{n}*\left( \frac{p_{t}}{1-p_{t}} \right),$$

where *n* is the sample size, for example, 810 (see Table S6). For instance, in Table S6, at $p_{t}$ = 1% (or 0.01), the NB of 0.0075 is obtained by

$$\frac{9}{810}-\frac{289}{810}*\left( \frac{0.01}{1-0.01} \right).$$

The NB of 0.0075 means that when using the prediction model to make a treatment decision (at the 1% threshold probability), there would be 7.5^[[1]](#footnote-1)^ *more* true positives among every 1,000 individuals receiving a prognosis, *without* an increase of false positives. In comparison, the treat all strategy achieves two thirds less (2.4, instead of 7.5, more true positives among 1,000 individuals).

**Why “net” benefit**

The word “net” in NB means that NB expresses whatever remains after the weighted harms (false positives) have been subtracted from the gross benefits (true positives). This is why NB is interpreted as how many *more* true positives are obtained *without* increasing false positives, at a given threshold probability. A negative NB or an NB that is smaller than the NB of an alternative decision strategy indicates a potentially harmful decision strategy, if used in clinical practice.

**Conclusion**

DCA is an empirical way of finding the best possible source of information for clinical decision making in the presence of uncertainty [2,3], about either the existence of a disease (diagnosis) or a future event (prognosis), such as a suicide attempt.

# **Section 5 - Calibration details**

In this section we present model performance results regarding the model’s calibration, not its clinical utility.


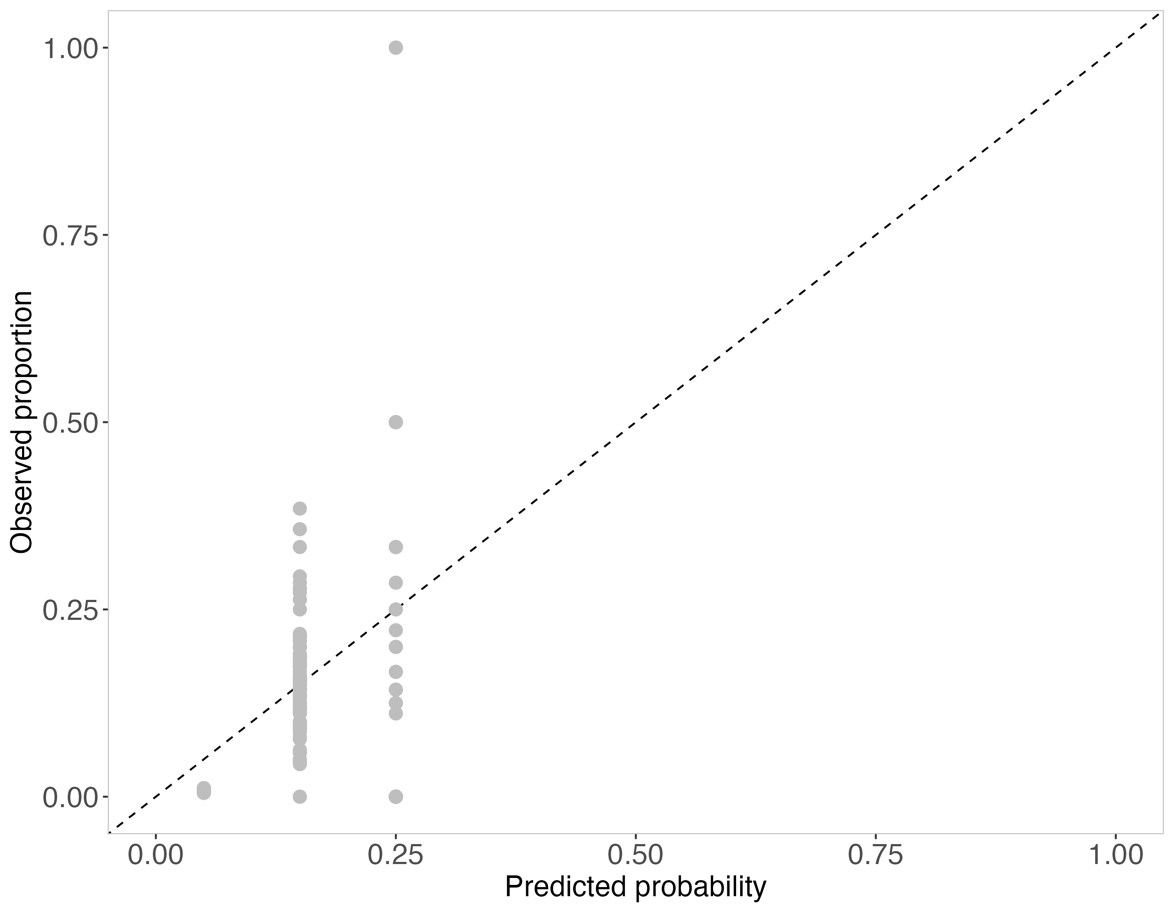


**Figure S3**. All of the 100 cross-validated calibration visualizations for the logistic regression model. Predicted probabilities ranged between 0.00007 and 0.296. In a well-calibrated model, the points align to the optimum, that is, the dashed diagonal, whereas the further away the points are from the diagonal, the more poorly the model is calibrated. Points above the dashed diagonal show an underestimation of the observed proportion of the outcome, whereas points below the dashed diagonal show an overestimation. Among the 100 cross-validated calibration visualizations, there were nine observed proportions beyond 0.4 (see *y* axis). That is, when the predicted probabilities were between 0.2 and 0.3 (20–30%), the observed proportion was 0.5 (50%) in five of these nine cases, and 1 (100%) in the remaining four cases. An observed proportion of 50% means that although the predicted probabilities in the range between 20 and 30% suggest that among all individuals who received a predicted probability between 0.2 and 0.3, somewhere around 25% of these individuals are expected to have the specified outcome (assuming a very well calibrated model), in fact 50% had the outcome (indicating a miscalibrated model in the range of predicted probabilities between 0.2 and 0.3).

NB can be good even for miscalibrated models, particularly if the model has the overall highest NB *and* is not showing concerning anomalies across the reasonable range of the prespecified threshold probabilities, i.e., increasing, instead of decreasing NB, as threshold probabilities increase, or a wide plateau of the model decision curve. The logic of this is that the calibration of a model is irrelevant if that miscalibration takes place beyond the a priori selected threshold probability (for which the NB is relevant). The same logic leads to the possibility of an overall well-calibrated model and/or a model with excellent discrimination, which nonetheless can be clinically harmful (have lower NB than competing decision alternatives) at the prespecified threshold probabilities.

Results of model calibration (based on 20% test subsets of each of the 100 model validations) are summarized with the Integrated Calibration Index (ICI), recently developed by Austin and Steyerberg [4]. According to its developers, the ICI is a calibration measure that explicitly incorporates the distribution of the predicted probabilities. The ICI developers state that the ICI (as well as a few other calibration metrics) allows for a greater differentiation in calibration, compared to what a visual inspection of graphical calibration curves permit. An ICI of exactly 0 denotes perfect calibration. In the 100 model validations in this study report, the ICI ranged between 0.003 and 0.018 (mean and median ICI = 0.008). For computation, we used the R code that was supplied in the Appendix in Austin and Steyerberg [4]. Results of the other performance metrics, from the model development perspective (i.e., discrimination and/or calibration), are presented in Table S7. We report these results according to transparent reporting recommendations. That is, these performance metrics were not our primary interest.

**Table S7**. Eight model development performance metrics and their cross-validated summary results across all 100 held-out test subsamples

| Metric | Min | 1st Qu. | Mean | Median | 3rd Qu. | Max |
| --- | --- | --- | --- | --- | --- | --- |
| Brier | 0.011 | 0.011 | 0.012 | 0.012 | 0.012 | 0.013 |
| Brier scaled | -0.037 | 0.032 | 0.048 | 0.050 | 0.070 | 0.134 |
| C. intercept | -2.658 | -0.697 | -0.155 | -0.302 | 0.349 | 2.594 |
| C. slope | 0.322 | 0.783 | 0.949 | 0.898 | 1.062 | 1.748 |
| ICI | 0.003 | 0.006 | 0.008 | 0.008 | 0.010 | 0.018 |
| E50 | 0.001 | 0.002 | 0.003 | 0.003 | 0.004 | 0.007 |
| E90 | 0.004 | 0.009 | 0.013 | 0.011 | 0.016 | 0.024 |
| E_max_ | 0.015 | 0.117 | 0.195 | 0.161 | 0.259 | 0.701 |

*Note*. Min = minimum; 1st Qu. = 25% quartile; 3rd Qu. = 75% quartile; Max = maximum, C. calibration. The Brier score, calibration intercept, integrated calibration index (ICI), E50, E90, and E_max_ all have the value 0 to represent the perfect result; the scaled Brier score and the calibration slope have the value 1 to represent the perfect result. The metrics E50, E90, and E_max_ represent the absolute difference between observed outcome rates and predicted probabilities, with E50 and E90 showing the median and 90th percentile and E_max_ showing the maximum absolute difference.

**Brier score and scaled Brier score**

The scaled Brier score permits better interpretation than the Brier score. The scaled Brier score is conceptually similar to Pearson’s $R^{2}$ statistic of a linear regression model [5]. That is, compared to a null model, which assigns the same overall risk to every individual, a scaled Brier score of 0.05 (see Table S7, median scaled Brier score) means that 5% of the prediction errors of the null model are explained by the prediction model. A scaled Brier score of 1.0 stands for the perfect model, whereas a negative scaled Brier score stands for a prediction model that shows even greater prediction errors than the null model.

**Calibration intercept and calibration slope**

A calibration intercept of less than 0 indicates an overestimation of risk predictions (all points below the diagonal overestimate the risk in Figure S3), whereas a value greater than 0 indicates an underestimation of risk predictions (all points above the diagonal underestimate the risk in Figure S3). A calibration slope of less than 1 indicates overly extreme risk predictions, whereas a value of greater than 1 indicates overly moderate risk predictions. It has been recommended to present detailed calibration results for models that generate individual risk predictions [6,7].

# **Section 6 - Logistic regression model output**

The logistic regression model output is shown in Table S8.

**Table S8.** Logistic regression model coefficients and odds ratios (OR)

|  | Coefficient | *SE* | OR | OR 95% CI |
| --- | --- | --- | --- | --- |
| Intercept | -4.0296 | 0.9735 | 0.02 | 0.003–0.12 |
| Lifetime SA | 2.5446 | 0.3096 | 12.74 | 6.94–23.37 |
| Lifetime mental disorder | 1.1711 | 0.5346 | 5.54 | 1.94–15.79 |
| Sex | -0.0631 | 0.3100 | 0.94 | 0.51–1.72 |
| Age | -0.0423 | 0.0165 | 0.96 | 0.93–0.99 |

*Note*. SA = suicide attempt; CI = confidence interval.

# **Section 7 - DCA details**

This section presents the details of the DCA.

**Table S9.** Net benefit (NB) and delta NB results. Top = median NB, middle = maximum NB, bottom = minimum NB.

| *p*_t_ % | Net benefit | | Median net benefit across 100 cross-validations | | | |
| --- | --- | --- | --- | --- | --- | --- |
|  |  |  | Logistic regression | | CART | |
|  | Treat all | Treat none | Total | Delta | Total | Delta |
| 0.00 | 0.0123 | 0 | 0.0123 | 0 | 0.0123 | 0 |
| 0.50 | 0.0074 | 0 | **0.0087** | 0.0013 | 0.0054 | -0.0020 |
| 0.75 | 0.0049 | 0 | **0.0078** | 0.0029 | 0.0056 | 0.0007 |
| 1.00 | 0.0024 | 0 | **0.0060** | 0.0037 | 0.0049 | 0.0026 |
| 1.25 | < 0 | 0 | **0.0046** | – | 0.0041 | – |
| 1.50 | < 0 | 0 | **0.0037** | – | 0.0034 | – |
| 1.75 | < 0 | 0 | **0.0036** | – | 0.0032 | – |
| 2.00 | < 0 | 0 | **0.0040** | – | 0.0028 | – |
| *p*_t_ % | Net benefit | | Maximum net benefit across 100 cross-validations | | | |
|  |  |  | Logistic regression | | CART | |
|  | Treat all | Treat none | Total | Treat all | Treat none | Delta |
| 0.00 | 0.0123 | 0 | 0.0123 | 0 | 0.0123 | 0 |
| 0.50 | 0.0074 | 0 | **0.0100** | 0.0027 | 0.0099 | 0.0025 |
| 0.75 | 0.0049 | 0 | **0.0095** | 0.0046 | 0.0093 | 0.0044 |
| 1.00 | 0.0024 | 0 | **0.0093** | 0.0070 | 0.0087 | 0.0063 |
| 1.25 | < 0 | 0 | **0.0093** | – | 0.0082 | – |
| 1.50 | < 0 | 0 | **0.0083** | – | 0.0080 | – |
| 1.75 | < 0 | 0 | 0.0078 | – | **0.0081** | – |
| 2.00 | < 0 | 0 | **0.0078** | – | 0.0069 | – |
| *p*_t_ % | Net benefit | | Minimum net benefit across 100 cross-validations | | | |
|  |  |  | Logistic regression | | CART | |
|  | Treat all | Treat none | Total | Delta | Total | Delta |
| 0.00 | 0.0123 | 0 | 0.0123 | 0 | 0.0123 | 0 |
| 0.50 | 0.0074 | 0 | **0.0050** | -0.0024 | 0.0014 | -0.0060 |
| 0.75 | 0.0049 | 0 | **0.0044** | -0.0004 | 0.0020 | -0.0029 |
| 1.00 | 0.0024 | 0 | **0.0023** | -0.0001 | 0.0014 | -0.0009 |
| 1.25 | < 0 | 0 | 0.0006 | – | **0.0009** | – |
| 1.50 | < 0 | 0 | 0.0004 | – | **0.0007** | – |
| 1.75 | < 0 | 0 | -0.0002 | -0.0002 | **0.0004** | – |
| 2.00 | < 0 | 0 | **0.0003** | – | 0.0001 | – |

*Note*. CART = classification and regression tree; the column *p*_t_ % contains the seven selected threshold probabilities; 0% was added to satisfy reporting guidelines for decision curve analysis. The *p*_t_ of 1.25% is larger than the outcome rate in the test data (0.0123 = 1.23%), yielding a negative net benefit (< 0) for treat all, as do all *p*_t_ > 1.25%. These cells of the table are empty because delta net benefit is equal to net benefit, according to our delta net benefit definition. Negative delta net benefit results were observed 18 times (logistic regression) and 119 times (CART) across 700 cross-validations, when compared to either treat all or treat none. The 18 logistic regression negative delta net benefit results occurred at *p*_t_ = 0.5% (14), 0.75% (2), 1% (1), and 1.75% (1). The 119 CART negative delta net benefit results occurred at *p*_t_ = 0.5% (75), 0.75% (38), and 1% (6).

**Visualization of the 100 cross-validated decision curves**

Whenever the lowest of the reasonable range of threshold probabilities is close to 0%, it is recommended to display the decision curve starting at 0%, that is, not to truncate the left of the threshold probability axis. It is also recommended to smooth the decision curves to some degree, to avoid artifacts that are produced by binning the predicted probabilities. Therefore, in Figures S4 and S5, the threshold probabilities 0.75%, 1.25%, and 1.75% were not considered. Of note, some methodologists argue in favor of smoothing (avoiding statistical artifacts when visualizing results), whereas others argue against it. We decided to show the unsmoothed data in the main document and have presented smoothed data in Figures S4 and S5 for the logistic regression and CART models, respectively.

**Detailed results**

We provide all details in the supplementary R package, which can be downloaded from this GitHub repository <https://github.com/mmiche/predictSuiattPsyCoLaus>. We want to draw the reader’s attention to the download instruction on GitHub, i.e., to load the package once it has been installed, execute this command: help(package='predictSuiattPsyCoLaus'), then either click on the link “User guides, package vignettes and other documentation”, or click on one of the five help pages, e.g., logreg700, which contains the 700 results from the logistic regression model, e.g., the cell sizes of the 2 by 2 table. Next, from the GitHub repository, see folder name rscriptsVignettes, download the R scripts. The script E_DisplayResults enables reproducing all net benefit results, in full detail, which have been summarized by using the median net benefit in the main paper.

For example, after the R package predictSuiattPsyCoLaus has been installed and loaded in a program that can execute R commands, open the R script E_DisplayResults.R. Run script lines 2-55, after which you possess all 700 net benefit results from the logistic regression and from the CART model.

The first of the 700 net benefit results from the logistic regression model is obtained by following the three steps that are involved in every net benefit computation, as described in the abstract of the main paper:

1. Weigh the false positives (440 individuals). As weight, use the odds of the selected risk threshold, which ranges in our paper between 0.5% and 2%. The odds of 0.5, yields 0.5/(100-0.5) = 0.5/99.5, which is approximately 0.005. Therefore, the result of this first step is 440*0.005 = 2.2.
2. Subtract 2.2 from the true positives (9 individuals): 9-2.2 = 6.8.
3. Divide 6.8 by the sample size (810 individuals): 6.8/810 = 0.008 (net benefit; rounded to three decimals).

**Logistic regression**


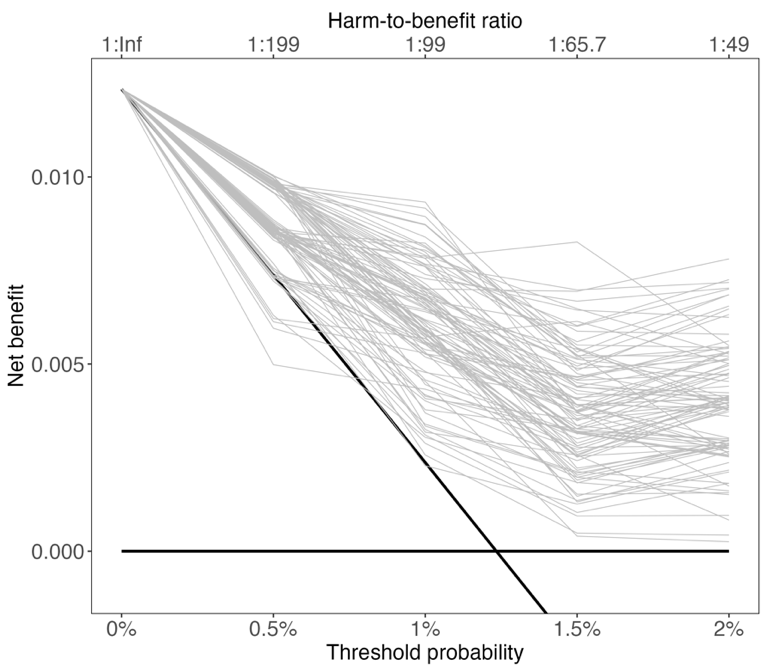


**Figure S4**. All of the 100 cross-validated decision curves for the logistic regression model, from which the net benefit performance measure is derived, using a priori selected threshold probabilities between 0.5% and 2%.

**CART model**


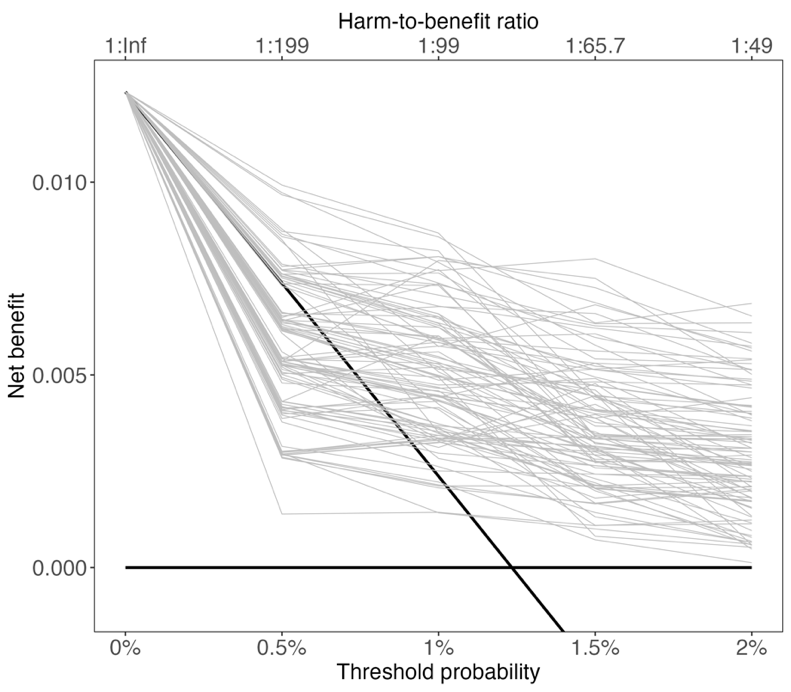


**Figure S5**. All of the 100 cross-validated decision curves for the classification and regression tree model, from which the net benefit performance measure is derived, using a priori selected threshold probabilities between 0.5% and 2%. The corresponding harm-to-benefit value was used as case weight during fitting the CART model to the data, e.g., 199 at the 0.5% threshold probability.

# **Section 8 - Development samples**

Each of the 100 distinct development samples consisted of 3240 individuals, of which 38 reported a suicide attempt at any of the three follow-up timepoints of the study. This yields an incidence rate of 0.0117 (1.17%). It is recommended to present the results regarding the development sample, in order to enable readers to evaluate the overfitting of a prediction model, when applied to the same data to which it was fitted, known as the development sample (i.e., the (training) subsample that was used to develop the prediction model).


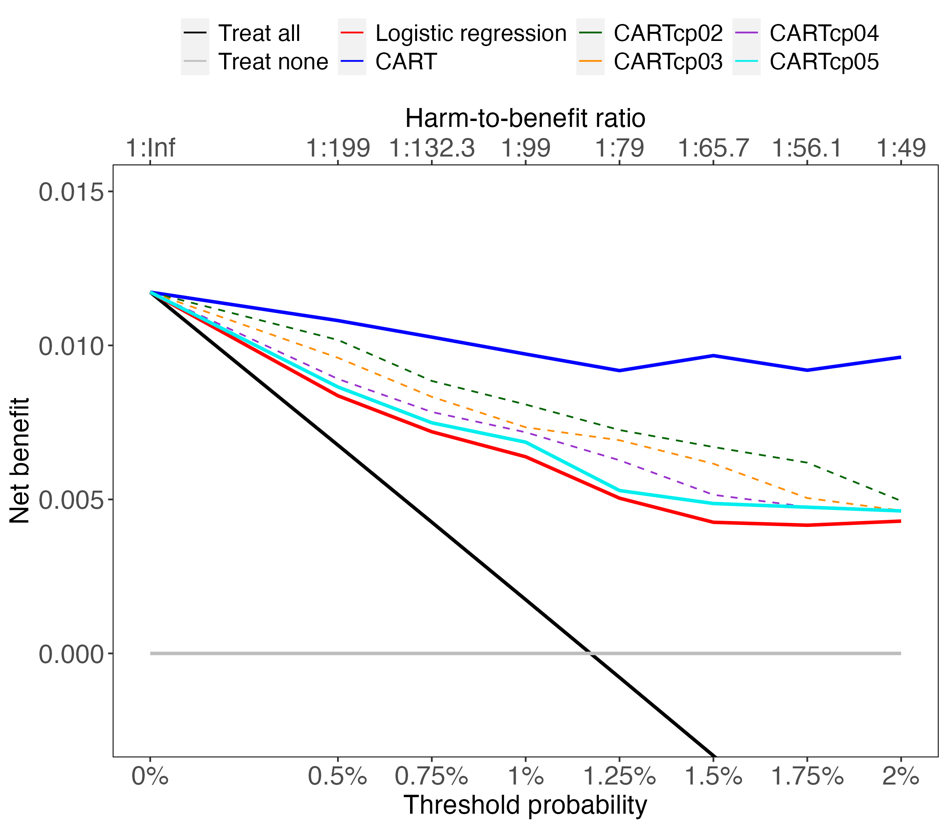


**Figure S6**. Decision curves for decision strategies treat all and treat none, and for six prediction models: Logistic regression (red solid), default CART (blue solid), and pruned CART (complexity parameter *c*_p_ between 0.02 and 0.05). Pruned CART curves for *c*_p_ = 0.02, 0.03, and 0.04 are shown as thin dashed curves between the solid blue and the solid cyan curve.

CART strongly overfitted, which can be seen when comparing the blue solid lines in Figure S1 and Figure S6. When pruning the overfitting CART model, the median NB closely approaches the median NB of the logistic regression model. The logistic regression model only slightly overfitted, which can be seen when comparing the red solid lines in Figure S1 and Figure S6.

**Table S10**. Median, maximum, and minimum NB results across the development subsamples (*N* = 3240)

|  | Net benefit | | | | | | | | | |
| --- | --- | --- | --- | --- | --- | --- | --- | --- | --- | --- |
| $p_{t}$% |  |  |  | Logistic regression | | |  | CART | | |
|  | Treat all | Treat none |  | Median | Max | Min |  | Median | Max | Min |
| 0.00 | 0.0117 | 0 |  | 0.0117 | 0.0117 | 0.0117 |  | 0.0117 | 0.0117 | 0.0117 |
| 0.50 | 0.0068 | 0 |  | 0.0084 | 0.0089 | 0.0079 |  | 0.0108 | 0.0112 | 0.0103 |
| 0.75 | 0.0043 | 0 |  | 0.0072 | 0.0083 | 0.0065 |  | 0.0103 | 0.0108 | 0.0094 |
| 1.00 | 0.0017 | 0 |  | 0.0064 | 0.0073 | 0.0054 |  | 0.0097 | 0.0106 | 0.0084 |
| 1.25 | < 0 | 0 |  | 0.005 | 0.0062 | 0.0041 |  | 0.0092 | 0.0104 | 0.0075 |
| 1.50 | < 0 | 0 |  | 0.0043 | 0.0057 | 0.003 |  | 0.0097 | 0.0105 | 0.0078 |
| 1.75 | < 0 | 0 |  | 0.0042 | 0.0054 | 0.0028 |  | 0.0092 | 0.0105 | 0.0052 |
| 2.00 | < 0 | 0 |  | 0.0043 | 0.0053 | 0.0028 |  | 0.0096 | 0.0103 | 0.0046 |

*Note*. CART = classification and regression tree; Max, maximum; Min, minimum; the column *p*_t_ % contains the seven selected threshold probabilities; 0% was added to satisfy reporting guidelines for decision curve analysis. The *p*_t_ of 1.25% is larger than the outcome rate in the development data (0.0117 = 1.17%), yielding a negative net benefit (< 0) for treat all, as do all *p*_t_ > 1.25%. These cells of the table are empty because delta net benefit is equal to net benefit, according to our delta net benefit definition.

**Table S11**. Four model development performance metrics and their cross-validated summary results across all 100 development subsamples

|  | Min | 1st Qu. | Mean | Median | 3rd Qu. | Max |
| --- | --- | --- | --- | --- | --- | --- |
| PR AUC | 0.0680 | 0.1111 | 0.1276 | 0.1272 | 0.1460 | 0.1843 |
| ROC AUC | 0.7944 | 0.8147 | 0.8256 | 0.8266 | 0.8347 | 0.8628 |
| ICI | 0.0011 | 0.0027 | 0.0037 | 0.0036 | 0.0043 | 0.0064 |
| Brier scaled | 0.0374 | 0.0569 | 0.0630 | 0.0633 | 0.0688 | 0.0874 |

*Note*. Min = minimum; 1st Qu. = 25% quartile; 3rd Qu. = 75% quartile; Max = maximum; PR AUC = area under the precision-recall curve; ROC AUC = area under the receiver operating characteristic curve; for both, a value of 1 represents perfect discrimination; ICI = Integrated Calibration Index; a value of 0 for the ICI represents perfect calibration.

Table S11 shows four performance results of the logistic regression model across the 100 development subsamples. Comparison with Table 4 in the main article and Table S7 reveals expected differences. However, similar to the median decision curve of the logistic regression model, when comparing Figures S6 and S1 (red solid lines), both mean and median results do not differ strongly. The strongest difference concerns the ICI (0.004 across the development subsamples versus 0.008 across the test subsamples).


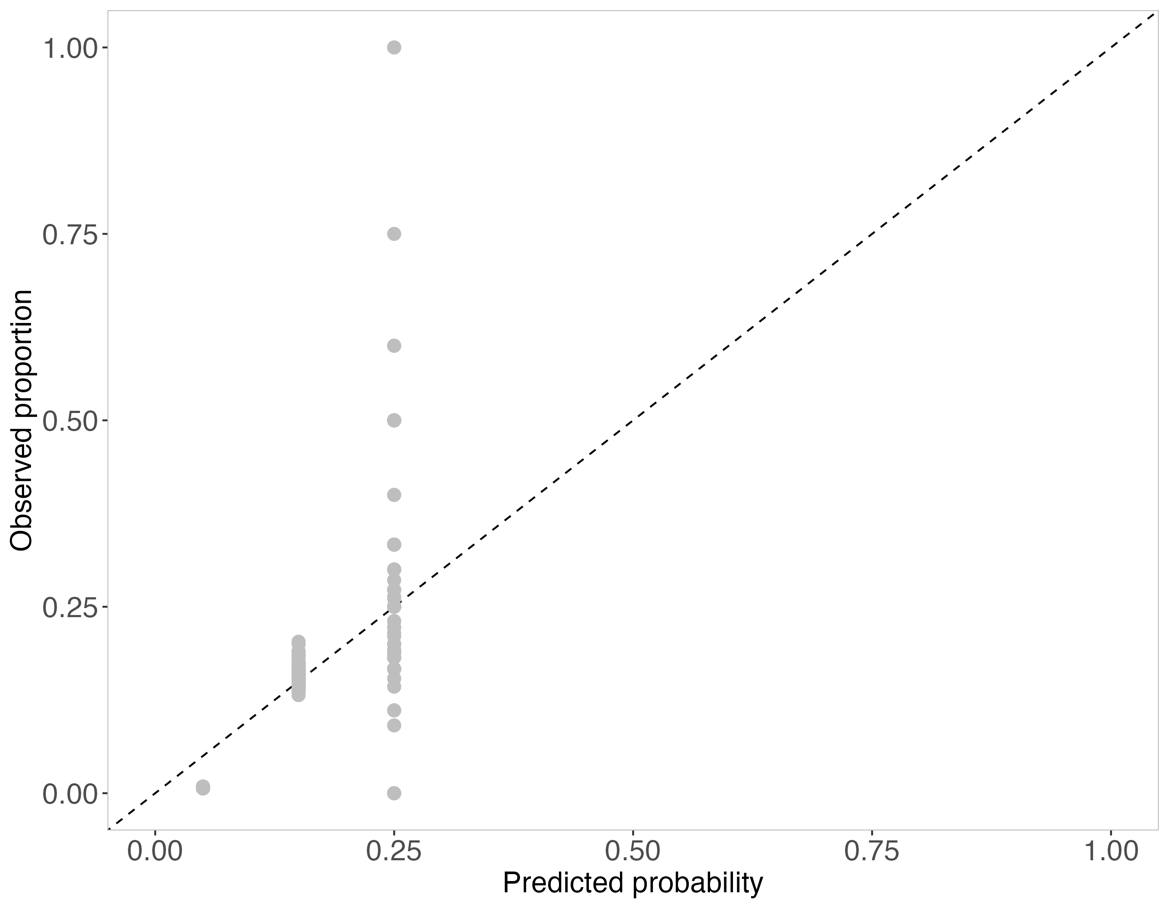


**Figure S7**. All of the 100 cross-validated calibration visualizations for the logistic regression model in the development subsample. Predicted probabilities ranged between 0.00007 and 0.291.

The comparison of Figure S7 and Figure S3 reveals a smaller spread in the observed proportion of individuals with the outcome (y-axis) for predicted probabilities between 10 and 20%, and a larger spread in the observed proportion for predicted probabilities between 20 and 30%.

# **References**

1. Therneau T, Atkinson B, Ripley B. rpart: Recursive partitioning and regression trees [Internet]. 2022. Available from: https://cran.r-project.org/web/packages/rpart/index.html

2. Pauker SG, Kassirer JP. Therapeutic decision making: A cost-benefit analysis. N Engl J Med [Internet]. 1975 Jul 31 [cited 2023 Apr 2];293(5):229–34. Available from: http://www.nejm.org/doi/abs/10.1056/NEJM197507312930505

3. Vickers AJ, Elkin EB. Decision curve analysis: A novel method for evaluating prediction models. Med Decis Making [Internet]. 2006 Nov [cited 2023 Apr 2];26(6):565–74. Available from: http://journals.sagepub.com/doi/10.1177/0272989X06295361

4. Austin PC, Steyerberg EW. The Integrated Calibration Index (ICI) and related metrics for quantifying the calibration of logistic regression models. Statistics in Medicine [Internet]. 2019 Sep 20 [cited 2023 Apr 3];38(21):4051–65. Available from: https://onlinelibrary.wiley.com/doi/10.1002/sim.8281

5. Steyerberg EW, Vickers AJ, Cook NR, Gerds T, Gonen M, Obuchowski N, et al. Assessing the performance of prediction models: A framework for traditional and novel measures. Epidemiology [Internet]. 2010;21(1):128–38. Available from: http://www.jstor.org/stable/25662818

6. Stevens RJ, Poppe KK. Validation of clinical prediction models: what does the “calibration slope” really measure? Journal of Clinical Epidemiology [Internet]. 2020 Feb [cited 2021 Jan 14];118:93–9. Available from: https://linkinghub.elsevier.com/retrieve/pii/S0895435619303579

7. Van Calster B, McLernon DJ, van Smeden M, Wynants L, Steyerberg EW, On behalf of Topic Group ‘Evaluating diagnostic tests and prediction models’ of the STRATOS initiative. Calibration: the Achilles heel of predictive analytics. BMC Med [Internet]. 2019 Dec [cited 2020 Jul 28];17(1):230. Available from: https://bmcmedicine.biomedcentral.com/articles/10.1186/s12916-019-1466-7

1. That is, 15 more true positives among every 2,000 individuals receiving a prognosis, since half individuals do not exist. [↑](#footnote-ref-1)
